# Impact of C-reactive protein on osteo-/chondrogenic transdifferentiation and calcification of vascular smooth muscle cells

**DOI:** 10.18632/aging.102130

**Published:** 2019-08-03

**Authors:** Laura A. Henze, Trang T.D. Luong, Beate Boehme, Jaber Masyout, Markus P. Schneider, Sebastian Brachs, Florian Lang, Burkert Pieske, Andreas Pasch, Kai-Uwe Eckardt, Jakob Voelkl, Ioana Alesutan

**Affiliations:** 1 Department of Internal Medicine and Cardiology, Charité – Universitätsmedizin Berlin, Campus Virchow-Klinikum, Berlin 13353, Germany; 2 Institute for Physiology and Pathophysiology, Johannes Kepler University Linz, Linz 4040, Austria; 3 Department of Nephrology and Hypertension, Friedrich-Alexander-Universität Erlangen-Nürnberg (FAU), 91054 Erlangen, Germany; 4 Department of Endocrinology, Diabetes and Nutrition, Charité - Universitätsmedizin Berlin, Campus Mitte, Berlin 10115, Germany; 5 DZHK (German Centre for Cardiovascular Research), partner site Berlin, Berlin 10115, Germany; 6 Department of Physiology I, Eberhard-Karls University, Tübingen 72076, Germany; 7 Berlin Institute of Health (BIH), Berlin 10178, Germany; 8 Department of Internal Medicine and Cardiology, German Heart Center Berlin (DHZB), Berlin 13353, Germany; 9 Calciscon AG, 2560 Nidau-Biel, Switzerland; 10 Department of Nephrology and Medical Intensive Care, Charité – Universitätsmedizin Berlin, Berlin 10117, Germany

**Keywords:** CRP, CKD, oxidative stress, vascular calcification, osteo-/chondrogenic signaling, vascular smooth muscle cells

## Abstract

Medial vascular calcification occurs during the aging process and is strongly accelerated by chronic kidney disease (CKD). Elevated C-reactive protein (CRP) levels are associated with vascular calcification, cardiovascular events and mortality in CKD patients. CRP is an important promoter of vascular inflammation. Inflammatory processes are critically involved in initiation and progression of vascular calcification. Thus, the present study explored a possible impact of CRP on vascular calcification. We found that CRP promoted osteo-/chondrogenic transdifferentiation and aggravated phosphate-induced osteo-/chondrogenic transdifferentiation and calcification of primary human aortic smooth muscle cells (HAoSMCs). These effects were paralleled by increased cellular oxidative stress and corresponding pro-calcific downstream-signaling. Antioxidants or p38 MAPK inhibition suppressed CRP-induced osteo-/chondrogenic signaling and mineralization. Furthermore, silencing of Fc fragment of IgG receptor IIa (FCGR2A) blunted the pro-calcific effects of CRP. Vascular CRP expression was increased in the klotho-hypomorphic mouse model of aging as well as in HAoSMCs during calcifying conditions. In conclusion, CRP augments osteo-/chondrogenic transdifferentiation of vascular smooth muscle cells through mechanisms involving FCGR2A-dependent induction of oxidative stress. Thus, systemic inflammation may actively contribute to the progression of vascular calcification.

## Introduction

Medial vascular calcification is characterized by the deposition of calcium phosphate in the media of arteries [1]. This process occurs during aging, but is strongly accelerated by chronic kidney disease (CKD), a condition of accelerated vascular aging [2,3]. The initiation and progression of vascular calcification is a complex process triggered by many pathological factors [4–6]. A key role in this process is attributed to vascular smooth muscle cells (VSMCs), which transdifferentiate into osteoblast- and chondroblast-like cells to promote vascular tissue mineralization [5,7,8]. The osteo-/chondrogenic phenotypical changes in VSMCs involve increased expression of osteogenic transcription factors and enzymes [5,7,9], paralleled by reduced expression of smooth muscle-specific proteins and, thus, loss of the contractile phenotype [10]. Osteo-/chondrogenic transdifferentiation of VSMCs and subsequent vascular calcification are tightly controlled by intracellular signaling pathways [5,8,9,11]. Various pathological factors activate pro-inflammatory signaling pathways to promote or augment VSMCs osteoinduction and calcification [12–15].

Pro-inflammatory plasma proteins include C-reactive protein (CRP) [16,17], synthesized mainly by hepatocytes during inflammatory or infectious processes [17,18], but also by other cell types including local production in vascular tissue by VSMCs [19,20]. Circulating CRP levels are affected by various factors such as age, blood pressure, obesity or smoking [17,21,22] and are a biomarker of inflammation [18]. Moreover, serum CRP concentrations are strongly associated with increased risk for development of atherosclerosis [20,23], cardiovascular disease [18,24–26] or hypertension [27] as well as death [25,28]. CRP is also able to induce pro-inflammatory responses in VSMCs [29,30].

Elevated serum CRP concentrations are associated with coronary calcium score in the elderly [31]. Increased CRP levels are also frequently observed in CKD patients [28,32] and are associated with disease progression [28]. Serum CRP concentrations are independent predictors of cardiovascular and all-cause mortality in these patients [33,34]. In CKD, the increased risk for cardiovascular events and high mortality may result to a large extent from vascular calcification [35]. In accordance, increased CRP concentrations are a risk factor associated with the development of vascular calcification [36,37]. CRP has been observed in uremic vascular tissue [38]. However, the direct impact of CRP on vascular calcification and the underlying mechanisms remained ill-defined.

The present study explored whether CRP promotes osteo-/chondrogenic transdifferentiation and calcification of VSMCs *in vitro* and, thus, contributes directly to the progression of vascular calcification.

## RESULTS

### Effects of CRP on osteo-/chondrogenic transdifferentiation of HAoSMCs

**Figure 1:**
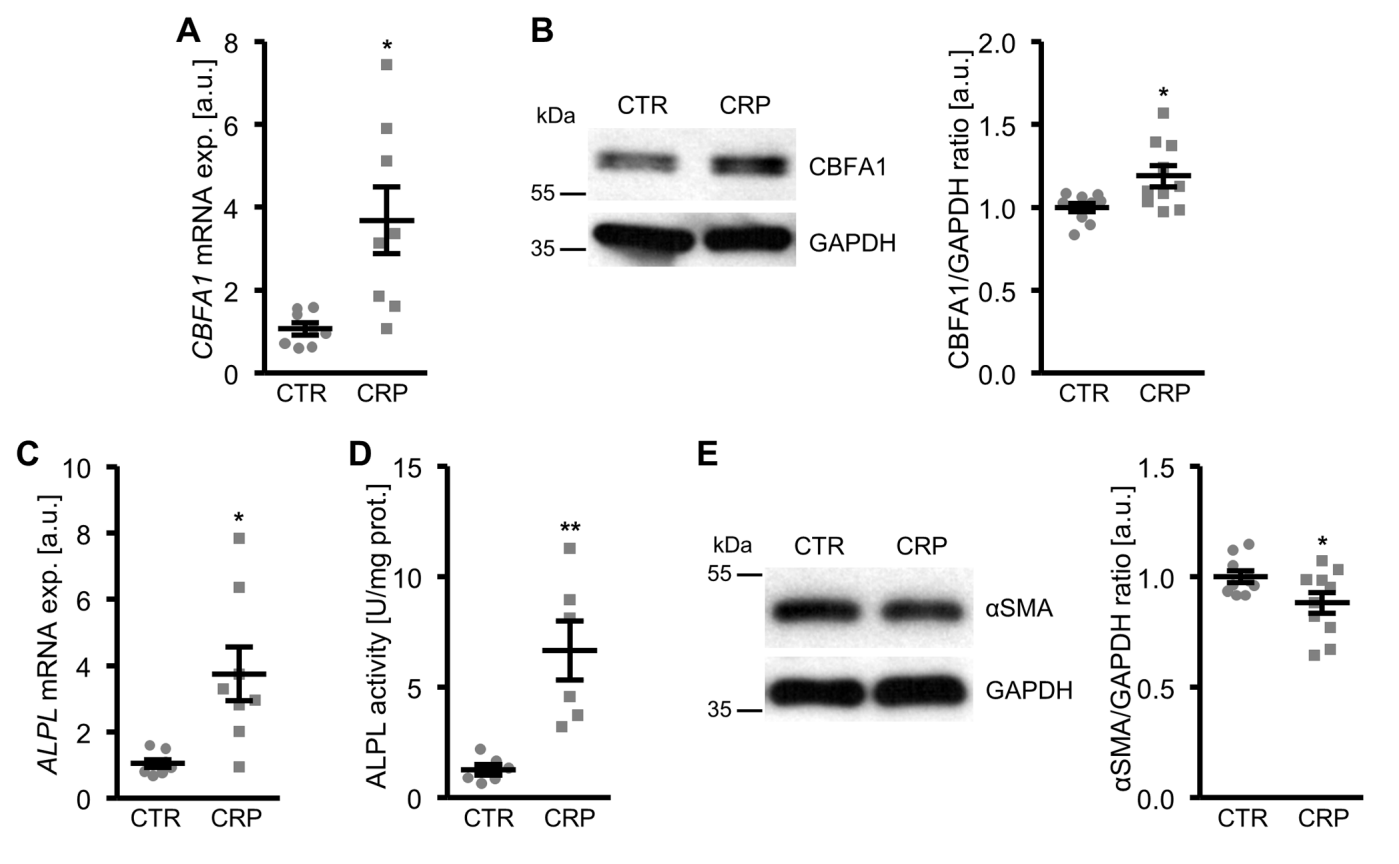
**CRP promotes osteo-/chondrogenic transdifferentiation of HAoSMCs.** (**A**) Scatter dot plots and arithmetic means ± SEM (n=8; arbitrary units, a.u.) of *CBFA1* relative mRNA expression in HAoSMCs treated with control (CTR) or 10 µg/ml recombinant human CRP. (**B**) Representative original Western blots and scatter dot plots and arithmetic means ± SEM (n=10; a.u.) of normalized CBFA1/GAPDH protein ratio in HAoSMCs treated with control (CTR) or 10 µg/ml recombinant human CRP. (**C**) Scatter dot plots and arithmetic means ± SEM (n=8; a.u.) of *ALPL* relative mRNA expression in HAoSMCs treated with control (CTR) or 10 µg/ml recombinant human CRP. (**D**) Scatter dot plots and arithmetic means ± SEM (n=6, U/mg protein) of ALPL activity in HAoSMCs treated with control (CTR) or 10 µg/ml recombinant human CRP. (**E**) Representative original Western blots and scatter dot plots and arithmetic means ± SEM (n=10; a.u.) of normalized αSMA/GAPDH protein ratio in HAoSMCs treated with control (CTR) or 10 µg/ml recombinant human CRP. *(p<0.05), **(p<0.01) significant vs. control HAoSMCs.

To determine whether CRP may contribute to vascular calcification, possible direct effects of CRP on osteo-/chondrogenic transdifferentiation of VSMCs were investigated. To this end, primary human aortic smooth muscle cells (HAoSMCs) were treated with recombinant human CRP. As a result, CRP up-regulated *CBFA1* mRNA and protein expression in HAoSMCs, an osteogenic transcription factor and marker of increased vascular osteoinduction (Figure 1A, B; Supplementary Figure 1). Similarly, CRP increased the expression and activity of the osteogenic enzyme tissue-nonspecific alkaline phosphatase (*ALPL*) (Figure 1C, D). These effects were paralleled by reduced protein levels of the smooth muscle-specific marker α-smooth muscle actin (αSMA) in CRP-treated HAoSMCs as compared to control-treated HAoSMCs (Figure 1E). Thus, CRP directly promoted osteo-/chondrogenic transdifferentiation of HAoSMCs.

### Effects of CRP on phosphate-induced osteo-/chondrogenic transdifferentiation and calcification of HAoSMCs

**Figure 2:**
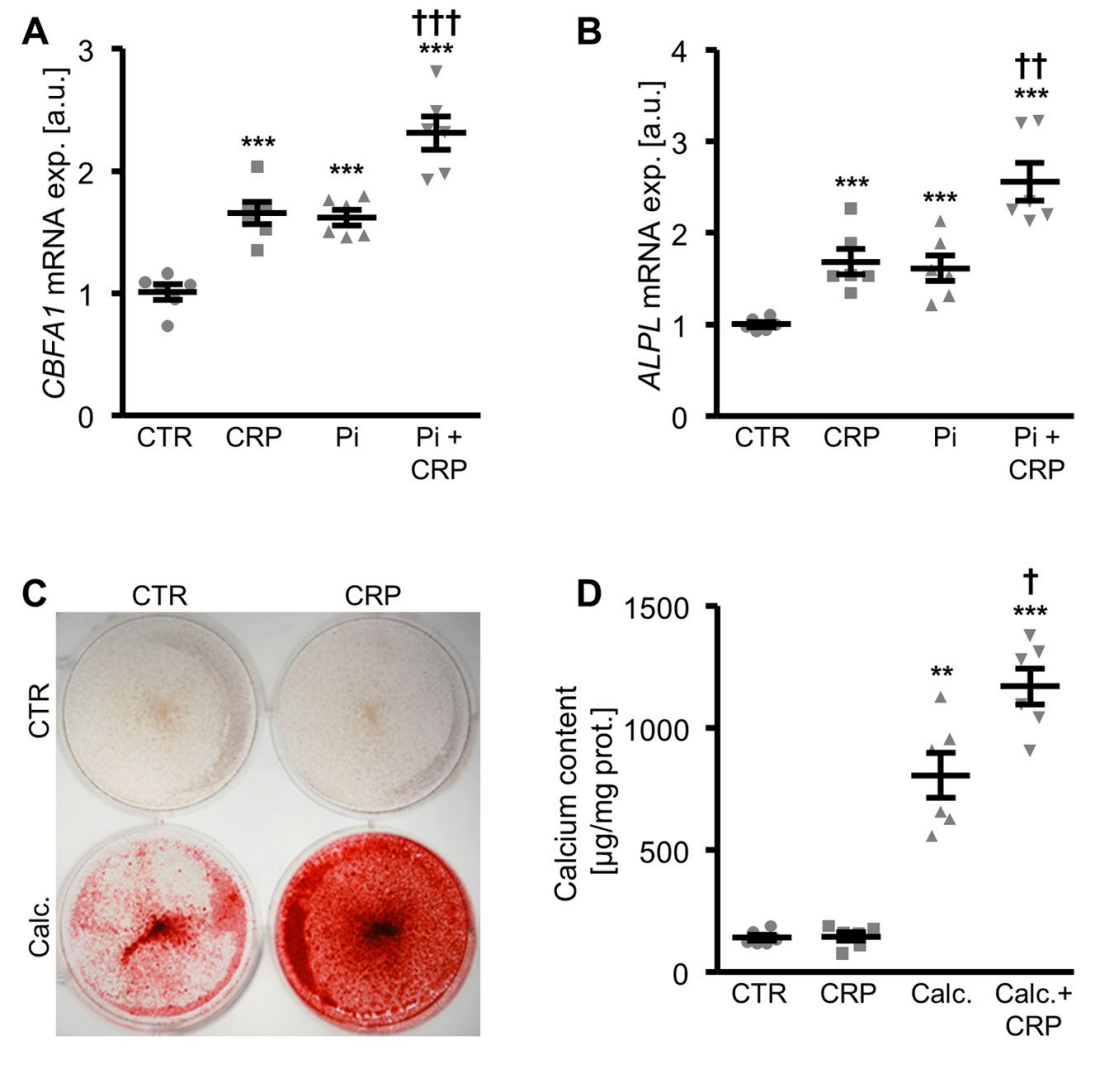
**CRP augments phosphate-induced osteo-/chondrogenic transdifferentiation and calcification of HAoSMCs.** (**A, B**) Scatter dot plots and arithmetic means ± SEM (n=6; arbitrary units, a.u.) of *CBFA1* (**A**) and *ALPL* (**B**) relative mRNA expression in HAoSMCs treated with control (CTR) or β-glycerophosphate (Pi) without and with 10 µg/ml recombinant human CRP. (**C**) Representative original images (n=4) showing Alizarin red staining in HAoSMCs treated with control (CTR) or calcification medium (Calc.) without and with 10 µg/ml recombinant human CRP. The calcified areas are shown as red staining. (**D**) Scatter dot plots and arithmetic means ± SEM (n=6; µg/mg protein) of calcium content in HAoSMCs treated with control (CTR) or calcification medium (Calc.) without and with 10 µg/ml recombinant human CRP. **(p<0.01), ***(p<0.001) significant vs. control HAoSMCs; †(p<0.05), ††(p<0.01), †††(p<0.001) significant vs. HAoSMCs treated with Pi/Calc. alone.

A next series of experiments investigated the pro-calcific effects of CRP in HAoSMCs during hyperphosphatemic conditions. As shown in Figure 2A, B, *CBFA1* and *ALPL* mRNA expression was significantly higher in HAoSMCs treated with phosphate together with CRP than in HAoSMCs exposed to high phosphate concentrations alone. Similarly, addition of CRP to the cell culture medium of HAoSMCs pre-exposed to hyperphosphatemic conditions significantly augmented osteogenic markers expression, and, thus, osteo-/chondrogenic transdifferentiation of HAoSMCs (Supplementary Figure 2). Furthermore, additional treatment with CRP aggravated the calcification of HAoSMCs induced by calcification medium (Figure 2C, D). Taken together, CRP aggravated osteo-/chondrogenic transdifferentiation and calcification of HAoSMCs during hyperphosphatemic conditions.

#### Effects of CRP on cellular oxidative stress and oxidative stress-downstream pro-calcific signaling in HAoSMCs

**Figure 3:**
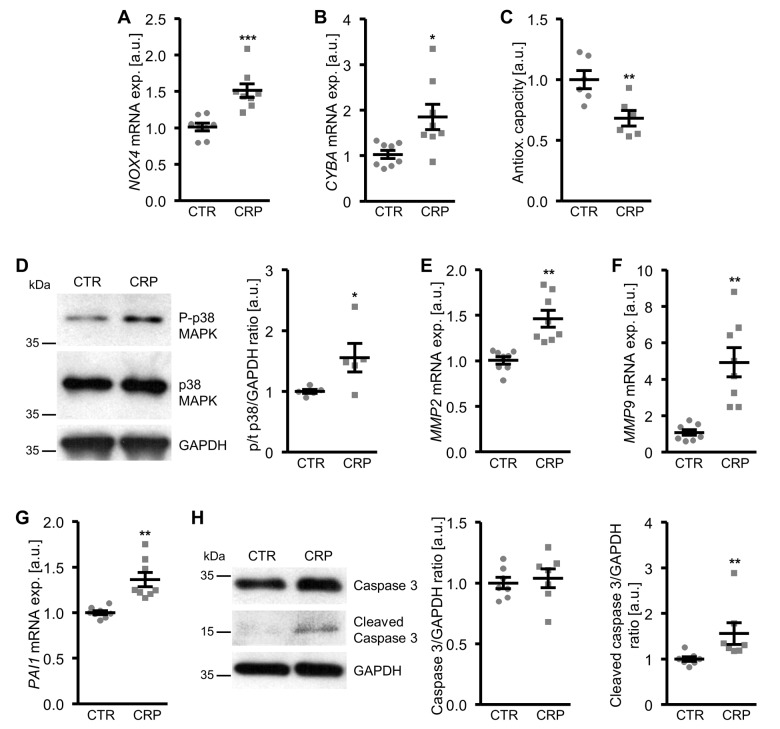
**CRP increases cellular oxidative stress and oxidative stress-downstream signaling in HAoSMCs.** (**A, B**) Scatter dot plots and arithmetic means ± SEM (n=8; arbitrary units, a.u.) of *NOX4* (**A**) and *CYBA* (**B**) relative mRNA expression in HAoSMCs treated with control (CTR) or 10 µg/ml recombinant human CRP. (**C**) Scatter dot plots and arithmetic means ± SEM (n=6; a.u.) of normalized total antioxidant capacity of HAoSMCs treated with control (CTR) or 10 µg/ml recombinant human CRP. (**D**) Representative original Western blots and scatter dot plots and arithmetic means ± SEM (n=5; a.u.) of normalized phospho-p38/total p38/GAPDH protein ratio in HAoSMCs treated with control (CTR) or 10 µg/ml recombinant human CRP. (**E-G**) Scatter dot plots and arithmetic means ± SEM (n=8; a.u.) of *MMP2* (**E**), *MMP9* (**F**) and *PAI1* (**G**) relative mRNA expression in HAoSMCs treated with control (CTR) or 10 µg/ml recombinant human CRP. (**H**) Representative original Western blots and scatter dot plots and arithmetic means ± SEM (n=7; a.u.) of normalized caspase 3/GAPDH and cleaved caspase 3/GAPDH protein ratio in HAoSMCs treated with control (CTR) or 10 µg/ml recombinant human CRP. *(p<0.05), **(p<0.01), ***(p<0.001) significant vs. control HAoSMCs.

To explore the underlying mechanisms involved in the pro-calcific action of CRP, the effects on cellular oxidative stress were investigated. As shown in Figure 3A-C, CRP treatment increased the mRNA expression of oxidative stress markers *NOX4* and *CYBA* (encoding p22phox) and decreased total antioxidant capacity and, thus, promoted oxidative stress in HAoSMCs.

Moreover, CRP increased the oxidative stress-downstream signaling leading to osteo-/chondrogenic phenotypical switch of HAoSMCs. CRP treatment activated the p38 MAPK signaling pathway, as shown by increased phosphorylation of p38 MAPK (Figure 3D). In contrast, SAPK/JNK MAPK and ERK1/2 MAPK pathways were not significantly affected by CRP treatment in HAoSMCs (data not shown). CRP up-regulated the mRNA expression of matrix gelatinases *MMP2* and *MMP9* (Figure 3E, F) as well as of plasminogen activator inhibitor *PAI1* (Figure 3G), downstream effectors of oxidative stress and important mediators of vascular calcification. In addition, the protein abundance of cleaved caspase 3 was higher in CRP-treated HAoSMCs than in control treated HAoSMCs (Figure 3H), pointing towards activated apoptotic signaling. Thus, CRP induced cellular oxidative stress and oxidative stress-downstream pro-calcific signaling in HAoSMCs.

### Impact of antioxidants and p38 MAPK inhibition on CRP-induced osteo-/chondrogenic signaling in HAoSMCs

**Figure 4:**
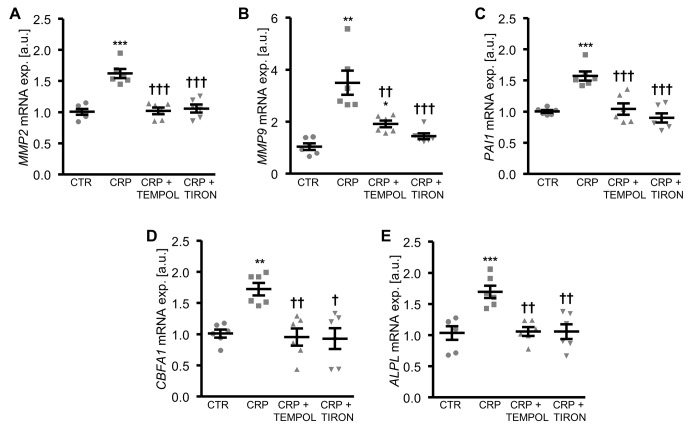
**Antioxidants suppress CRP-induced osteogenic signaling in HAoSMCs.** (**A-E**) Scatter dot plots and arithmetic means ± SEM (n=6; arbitrary units, a.u.) of *MMP2* (**A**), *MMP9* (**B**), *PAI1* (**C**), *CBFA1* (**D**) and *ALPL* (**E**) relative mRNA expression in HAoSMCs treated with control (CTR) or 10 µg/ml recombinant human CRP without and with 10 µM TEMPOL or 10 µM TIRON. *(p<0.05), **(p<0.01), ***(p<0.001) significant vs. control HAoSMCs; †(p<0.05), ††(p<0.01), †††(p<0.001) significant vs. HAoSMCs treated with CRP alone.

To further explore the involvement of oxidative stress in CRP-induced osteo-/chondrogenic transdifferentiation, HAoSMCs were treated with CRP in the presence or absence of the antioxidants TEMPOL or TIRON. As illustrated in Figure 4, antioxidants blunted the increase of *MMP2*, *MMP9* and *PAI1* as well as osteogenic *CBFA1* and *ALPL* mRNA expression following CRP treatment of HAoSMCs. Thus, oxidative stress mediated, at least in part, the pro-calcific effects of CRP in HAoSMCs.

**Figure 5:**
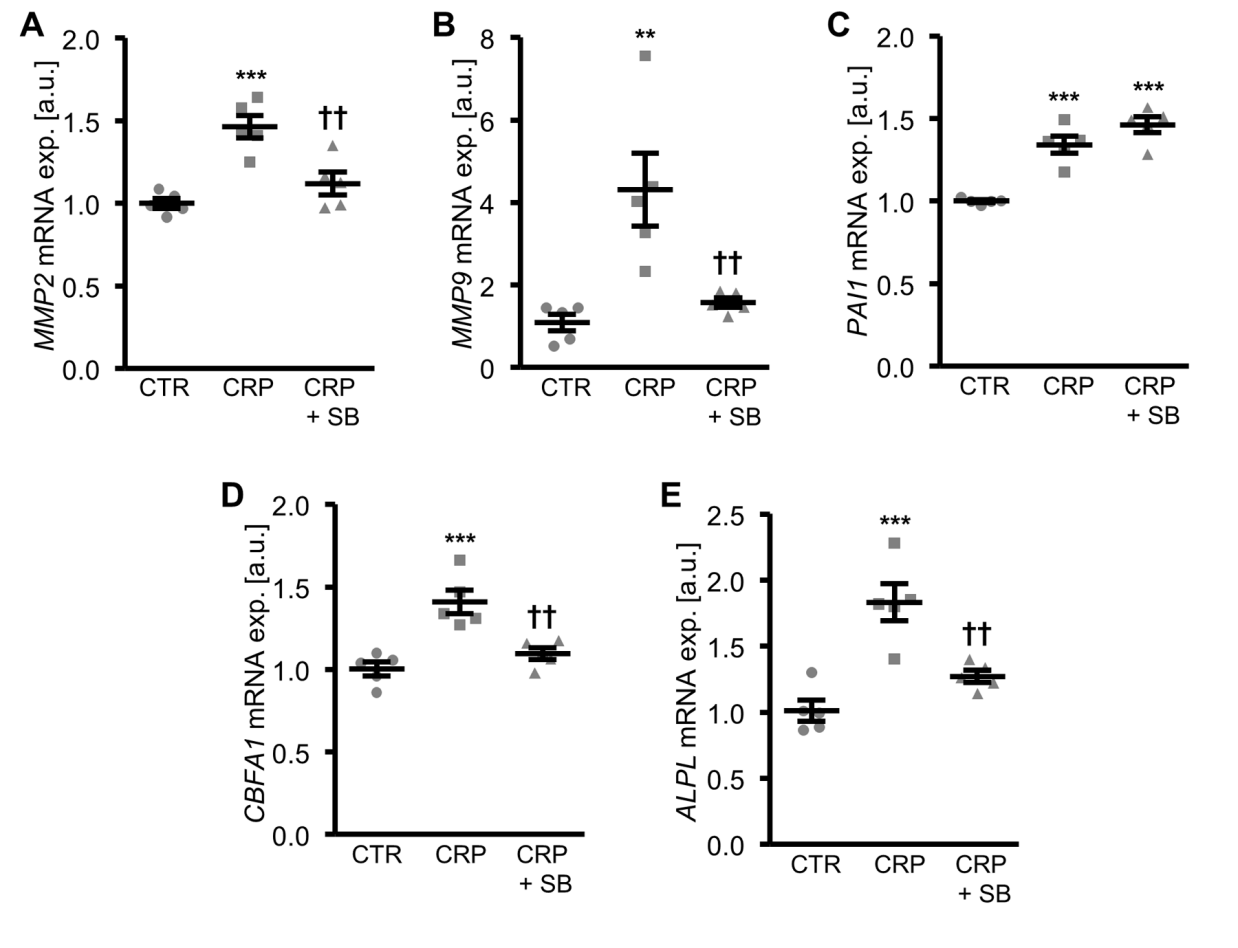
**Inhibition of p38 MAPK blunts CRP-induced osteogenic signaling in HAoSMCs.** (**A-E**) Scatter dot plots and arithmetic means ± SEM (n=5; arbitrary units, a.u.) of *MMP2* (**A**), *MMP9* (**B**), *PAI1* (**C**), *CBFA1* (**D**) and *ALPL* (**E**) relative mRNA expression in HAoSMCs treated with control (CTR) or 10 µg/ml recombinant human CRP without and with 10 µM p38 MAPK inhibitor SB203580 (SB). **(p<0.01), ***(p<0.001) significant vs. control HAoSMCs; ††(p<0.01) significant vs. HAoSMCs treated with CRP alone.

Inhibition of p38 MAPK signaling by pretreatment of HAoSMCs with the specific p38 MAPK inhibitor SB203580, blunted the CRP-induced mRNA expression of matrix gelatinases (Figure 5A, B) and osteogenic markers (Figure 5D, E). However, the inhibitor did not affect the increased *PAI1* mRNA expression in CRP-treated HAoSMCs (Figure 5C), suggesting that CRP-induced oxidative stress-dependent *PAI1* up-regulation was independent from the p38 MAPK signaling pathway.

**Figure 6:**
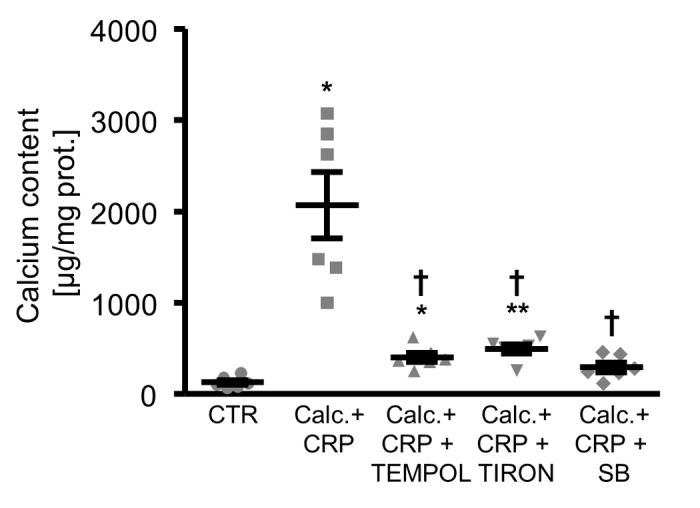
**Antioxidants or p38 MAPK inhibition reduce calcification of HAoSMCs promoted by CRP during pro-calcific conditions.** Scatter dot plots and arithmetic means ± SEM (n=6; µg/mg protein) of calcium content in HAoSMCs treated with control (CTR) or calcification medium together with 10 µg/ml recombinant human CRP (Calc.+CRP) and without and with additional treatment with 10 µM TEMPOL, 10 µM TIRON or 10 µM p38 MAPK inhibitor SB203580 (SB). *(p<0.05), **(p<0.01) significant vs. control HAoSMCs; †(p<0.05) significant vs. HAoSMCs treated with Calc.+CRP alone.

Moreover, antioxidants or p38 MAPK inhibition were all able to reduce the calcification of HAoSMCs promoted by CRP in the presence of calcification medium (Figure 6).

### Role of Fc fragment of IgG receptor IIa in CRP-induced oxidative stress and osteo-/chondrogenic signaling in HAoSMCs

**Figure 7:**
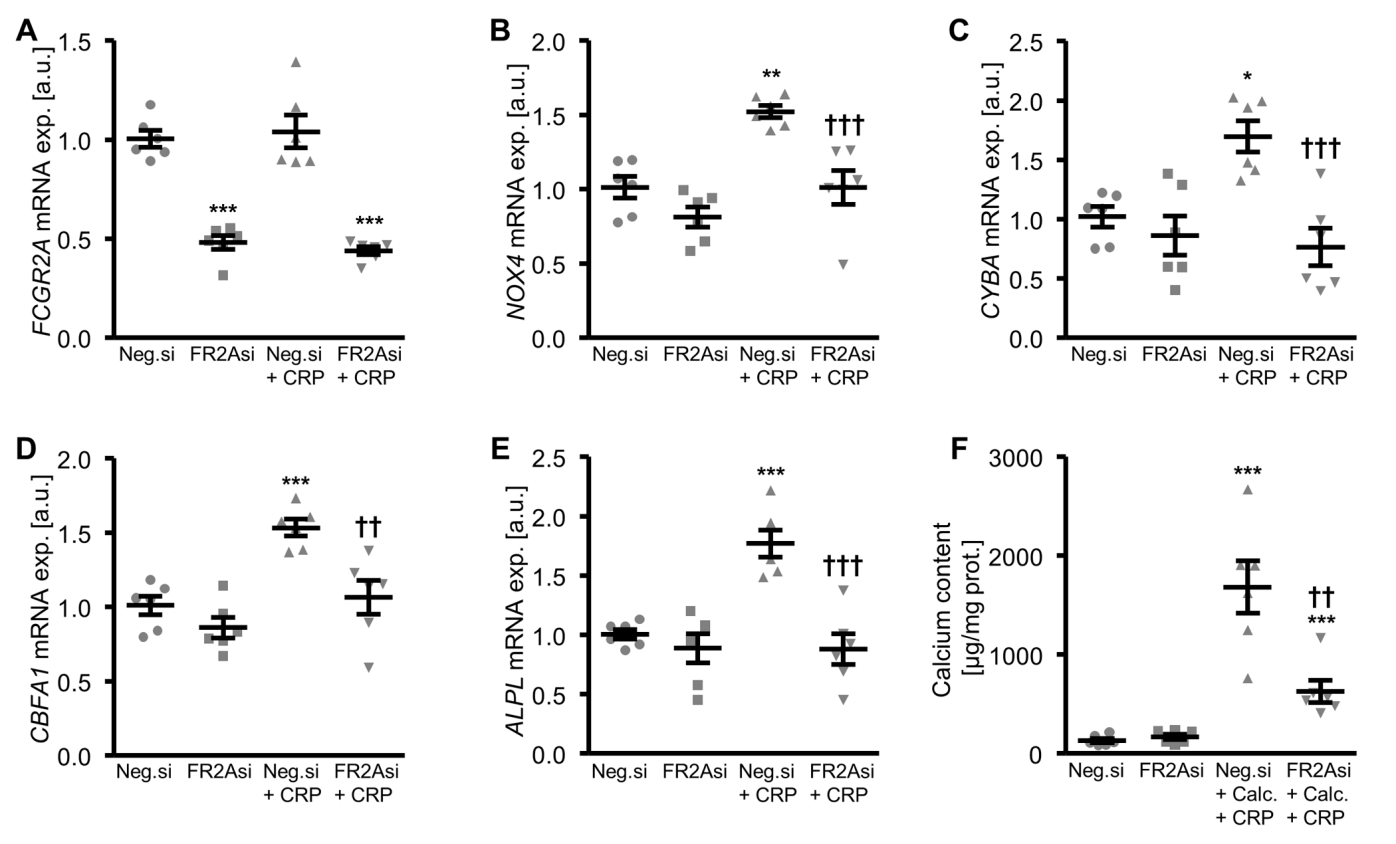
**Silencing of FCGR2A inhibits CRP-induced osteogenic signaling and calcification of HAoSMCs. **(**A-E**) Scatter dot plots and arithmetic means ± SEM (n=6; arbitrary units, a.u.) of *FCGR2A* (**A**), *NOX4* (**B**), *CYBA* (**C**), *CBFA1* (**D**) and *ALPL* (**E**) relative mRNA expression in HAoSMCs silenced with negative control siRNA (Neg.si) or FCGR2A siRNA (FR2Asi) and treated with control or 10 µg/ml recombinant human CRP. (**F**) Scatter dot plots and arithmetic means ± SEM (n=6; µg/mg protein) of calcium content in HAoSMCs silenced with negative control siRNA (Neg.si) or FCGR2A siRNA (FR2Asi) and treated with control or calcification medium together with 10 µg/ml recombinant human CRP (Calc.+CRP). *(p<0.05), **(p<0.01), ***(p<0.001) significant vs. Neg.si silenced HAoSMCs; ††(p<0.01), †††(p<0.001) significant vs. Neg.si silenced and CRP/Calc.+CRP treated HAoSMCs.

Further experiments investigated the role of Fc fragment of IgG receptor IIa (encoded by the *FCGR2A* gene) in CRP-dependent osteoinduction in HAoSMCs by silencing of the receptor using small interfering RNA (siRNA). *FCGR2A* expression was significantly lower in FCGR2A-silenced HAoSMCs as compared to HAoSMCs transfected with negative control siRNA (Figure 7A, Supplementary Figure 3). CRP treatment did not significantly alter *FCGR2A* mRNA expression in HAoSMCs (Figure 7A). The CRP-induced oxidative stress marker mRNA expression was blunted by silencing of FCGR2A in HAoSMCs (Figure 7B, C). Moreover, silencing of FCGR2A was sufficient to blunt the increase of osteogenic marker mRNA expression following CRP treatment of HAoSMCs (Figure 7D, E). Similarly, FCGR2A knockdown reduced the calcification of HAoSMCs induced by CRP and calcification medium (Figure 7F). Thus, CRP induced cellular oxidative stress, osteo-/chondrogenic transdifferentiation and calcification of HAoSMCs, at least partly, via the FCGR2A receptor.

### Vascular CRP expression during pro-calcifying conditions

**Figure 8:**
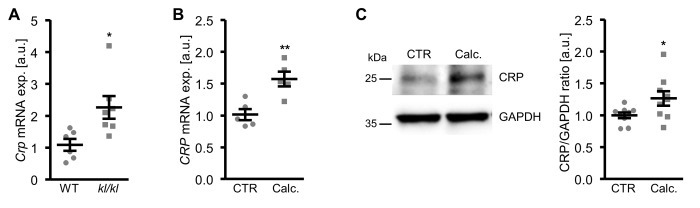
**Vascular CRP expression is increased during pro-calcifying conditions.** (**A**) Scatter dot plots and arithmetic means ± SEM (n=6-7; arbitrary units, a.u.) of *Crp* relative mRNA expression in aortic tissue of klotho-hypomorphic (*kl/kl*) mice and corresponding wild-type (WT) mice. (**B**) Scatter dot plots and arithmetic means ± SEM (n=5; a.u.) of *CRP* relative mRNA expression in HAoSMCs treated with control (CTR) or calcification medium (Calc.). (**C**) Representative original Western blots and scatter dot plots and arithmetic means ± SEM (n=9; a.u.) of normalized CRP/GAPDH protein ratio in HAoSMCs treated with control (CTR) or calcification medium (Calc.). *(p<0.05), **(p<0.01) significant vs. WT mice or control HAoSMCs, respectively.

In a next series of experiments, vascular CRP expression during pro-calcifying conditions was determined. As shown in Figure 8A, *Crp* mRNA expression was significantly higher in aortic tissue of hyperphosphatemic klotho-hypomorphic (*kl/kl*) mice as compared to corresponding wild-type mice, a model of premature aging and CKD-related vascular calcification.

Furthermore, *CRP* mRNA and protein expression were significantly up-regulated in HAoSMCs following treatment with calcification medium (Figure 8B, C). Aldosterone, a known factor influencing serum CRP levels and a trigger of vascular calcification did not significantly affect *CRP* mRNA expression (Supplementary Figure 4A) and did not have additive effects on osteogenic markers expression (Supplementary Figure 4B, C) in HAoSMCs.

### Association of serum CRP levels with serum calcification propensity in human CKD patients

To confirm that serum CRP levels associate with vascular calcification, the correlation with serum calcification propensity was determined in CKD patients. As a result, serum CRP concentrations inversely correlated with calciprotein particle maturation time (T_50_) and, thus, significantly associated with serum calcification propensity in the CARVIDA cohort of patients with moderately severe CKD (n=309; Spearman r -0.1344, p=0.0181).

## DISCUSSION

The present study discloses a novel direct role of CRP during vascular calcification. CRP induces cellular oxidative stress and activates pro-calcific intracellular signaling pathways promoting osteo-/chondrogenic transdifferentiation of VSMCs *in vitro*.

Osteo-/chondrogenic transdifferentiation of VSMCs plays a key role in initiation and progression of vascular calcification [6,8,39,40]. The complex osteoinductive signaling cascades in VSMCs eventually lead to up-regulation of osteogenic transcription factors such as CBFA1, with an essential role in vascular calcification [8]. CBFA1 deficiency blocks mineralization of VSMCs [41]. Further, the osteogenic transcription factors ultimately lead to up-regulation of ALPL, which degrades the endogenous calcification inhibitor pyrophosphate to allow unrestrained mineralization [8,42]. ALPL has therefore been considered a key effector during vascular calcification [8,42]. Here we show that CRP treatment leads to increased expression of the osteogenic markers CBFA1 and ALPL and reduces the levels of smooth muscle-specific markers, and, thus, promotes the phenotypical change of VSMCs into osteoblast-like cells. These effects further influence the calcification of VSMCs, as the mineral deposition induced during calcifying conditions [43,44] is augmented in the presence of CRP.

CRP contributes to vascular calcification by inducing oxidative stress in VSMCs. Oxidative stress may lead to osteo-/chondrogenic transdifferentiation of VSMCs [45,46] and is associated with vascular calcification in CKD [47]. Similar to findings in other cell types [48,49], CRP increases the expression of NOX4 and p22phox, components of the superoxide-generating NAPDH oxidase system that, in turn, is also associated with vascular calcification [50]. CRP-induced oxidative stress and subsequent pro-inflammatory responses in VSMCs require the Fc fragment of IgG type IIa (FCGR2A) receptor and NOX4 activation [29]. FCGR2A is a cell surface membrane receptor for IgG, CRP as well as serum amyloid P component [51], with an important role in inflammatory cardiovascular disorders [51–56]. In accordance, FCGR2A knockdown in VSMCs is able to block CRP-induced expression of oxidative stress and osteogenic markers as well as calcification, suggesting that the pro-calcific effects of CRP in VSMCs are FCGR2A-dependent.

Oxidative stress induces osteo-/chondrogenic transdifferentiation of VSMCs through several downstream signaling pathways [44–46]. The p38 MAPK pathway [46] plays a key role in controlling vascular calcification [46,57]. Moreover, oxidative stress may increase the expression of matrix gelatinases in VSMCs [45] and, thus, promote degradation of extracellular matrix to allow mineralization [58]. In VSMCs, CRP is able to activate p38 MAPK [59] and to increase the expression of matrix gelatinases [60]. The p38 MAPK mediates pro-inflammatory responses triggered by CRP in VSMCs [59]. Accordingly, the present findings show that CRP activates p38 MAPK in VSMCs, while inhibition of p38 MAPK pathway suppresses matrix gelatinases expression, osteo-/chondrogenic transdifferentiation and calcification of VSMCs. In addition, CRP induces the expression of plasminogen activator inhibitor PAI1 [61] through oxidative stress in VSMCs in a p38-independent manner. Activation of PAI1 by oxidative stress has been described previously [62]. PAI1 levels are increased in chronic inflammatory conditions associated with CKD [63]. PAI1 may promote senescence [64] and pro-inflammatory responses [65] and is considered a regulator of vascular calcification [4,45]. CRP further activates apoptotic signaling in VSMCs that may also contribute to vascular mineralization, as apoptotic bodies could serve as nidus sites for calcium phosphate precipitation [1].

Taken together, CRP promotes oxidative stress and oxidative stress-dependent osteoinductive signaling in VSMCs and induces osteo-/chondrogenic transdifferentiation of VSMCs and, therefore, may contribute directly to vascular calcification. Antioxidants or inhibition of the p38 MAPK pathway are sufficient to ameliorate the pro-calcific effects of CRP in VSMCs and, thus, are key elements in this osteoinductive signaling pathway. However, the present observations do not rule out involvement of other mechanisms besides oxidative stress.

VSMCs express CRP and, thus, CRP is also produced locally in the vasculature [19,66–68]. The present findings show that CRP expression is increased in VSMCs during calcifying conditions *in vitro* as well as in the vascular tissue of the klotho-hypomorphic mouse model of aging and CKD-related vascular calcification. In VSMCs, CRP expression may also be induced by various pathological factors including aldosterone [19] or homocysteine [68], known triggers of vascular calcification in CKD [6,7,69–72]. However, in our *in vitro* model, aldosterone did not significantly influence *CRP* expression or CRP-induced osteo-/chondrogenic transdifferentiation of VSMCs. In addition, cellular oxidative stress [19,66–68] and p38 MAPK pathway activation [66,67] may further augment CRP expression in VSMCs and, thus, increase local CRP concentrations leading to an amplification of the pro-calcific effects. Thus, in complex pathological conditions such as CKD, elevated systemic CRP levels as well as locally produced CRP may directly contribute to the progression of vascular calcification. Further studies are required to confirm the pro-calcific effects of CRP *in vivo* and the potential role of vascular CRP in vascular calcification.

The pro-calcific effects of CRP in VSMCs contributing to development of vascular calcification may further indirectly impact on the cardiovascular system. Vascular calcification may trigger an increase of arterial stiffness and pulse pressure, leading to microcirculatory dysfunction, impaired organ perfusion, cardiac hypertrophy and diastolic dysfunction [3,13,39]. The altered microenvironment in the vascular wall during vascular calcification may promote endothelial dysfunction [73,74], atherogenesis [75] as well as atherosclerotic plaque calcification and instability [3,76]. Thus, in addition to already described direct effects [17,18,77,78], the pro-calcific effect of CRP may indirectly contribute to other pathological alterations in the vasculature.

Serum CRP levels emerged as a surrogate marker of the inflammatory status [79]. Elevated serum CRP levels [28] and inflammation [12] are prevalent in CKD and are associated with the development of vascular calcification [12,36,37,80,81]. In accordance with previous studies [82,83], the present observations show that serum CRP concentrations correlate with serum calcification propensity and, thus, may be associated with an increased risk for vascular calcification in patients with CKD. However, to which extent the observed direct effect of CRP on VSMC calcification contributes to the pro-calcific phenotype requires further study.

In conclusion, CRP triggers osteo-/chondrogenic transdifferentiation of VSMCs and augments mineralization *in vitro*, at least partly, via FCGR2A-dependent induction of cellular oxidative stress. Thus, the present observations identified CRP as a new pathologic factor contributing to the progression of vascular calcification.

## MATERIALS AND METHODS

### Primary human aortic smooth muscle cells

Primary human aortic smooth muscle cells (HAoSMCs; Thermo Fisher Scientific) were used in all experiments from passages 4 to 12 [14,45,84]. HAoSMCs were cultured in Waymouth’s MB 752/1 medium and Ham’s F-12 nutrient mixture (1:1; Thermo Fisher Scientific) containing 10% FBS (Thermo Fisher Scientific), 100 U/ml penicillin and 100 µg/ml streptomycin (Thermo Fisher Scientific) [69]. At confluence, HAoSMCs were split into 6-well plates (2x10^5^ cells/well) and allowed to attach for 24 hours prior to treatment for the indicated times with 10 µg/ml recombinant human CRP protein (R&D Systems) [85,86], 10 µM TEMPOL (4-hydroxy-TEMPO, stock in DMSO; Sigma-Aldrich), 10 µM TIRON (4,5-dihydroxy-1,3-benzenedisulfonic acid disodium salt monohydrate; Sigma-Aldrich) [4,44], 10 µM p38 MAPK inhibitor SB203580 (stock in DMSO; Cayman Chemical) [57] or 100 nM aldosterone (stock in DMSO; Sigma-Aldrich) [9,69]. Equal amounts of vehicle were used as control. HAoSMCs were treated for 11 days with calcification medium containing 10 mM β-glycerophosphate and 1.5 mM CaCl_2_ (Sigma-Aldrich) [9,43,44,87]. Fresh media with agents were added every 2-3 days. HAoSMCs were transfected with 10 nM FCGR2A siRNA (ID no. s223524, Thermo Fisher Scientific) or 10 nM negative control siRNA (ID no. 4390843, Thermo Fisher Scientific) using siPORT amine transfection agent (Thermo Fisher Scientific) [44,70,88].

### Animal experiments

All animal experiments were conducted according to the recommendations of the Guide for Care and Use of Laboratory Animals of the National Institutes of Health as well as the German law for the welfare of animals, and reviewed and approved by the local government authority. The klotho-hypomorphic (*kl/kl*) mice and corresponding wild-type (WT) mice were previously described [84,89]. Mice were sacrificed in isoflurane anesthesia and aortic tissues were snap frozen in liquid nitrogen.

### Quantitative RT-PCR

Total RNA was isolated from HAoSMCs 48 hours and 24 hours following silencing and/or treatments, respectively and from murine aortic tissues by using Trizol Reagent (Thermo Fisher Scientific) [11,69]. cDNA was synthesized by using oligo(dT)_12-18_ primers (Thermo Fisher Scientific) and SuperScript III Reverse Transcriptase (Thermo Fisher Scientific). Quantitative RT-PCR was performed in duplicate with iQ^TM^ Sybr Green Supermix (Bio-Rad Laboratories) and CFX96 Real-Time PCR Detection System (Bio-Rad Laboratories). The specificity of the PCR products was confirmed by analysis of the melting curves. Relative mRNA expression was calculated by the 2^-ΔΔCt^ method using GAPDH as housekeeping gene, normalized to the control group.

The following human primers were used (Thermo Fisher Scientific, 5’→3’ orientation) [44,45,90]:

*ALPL* fw: GGGACTGGTACTCAGACAACG;

*ALPL* rev: GTAGGCGATGTCCTTACAGCC;

*CBFA1* fw: GCCTTCCACTCTCAGTAAGAAGA;

*CBFA1* rev: GCCTGGGGTCTGAAAAAGGG;

*CYBA* fw: CCCAGTGGTACTTTGGTGCC;

*CYBA* rev: GCGGTCATGTACTTCTGTCCC;

*CRP* fw: AACGAAGCCTCTCAAAGCCTT;

*CRP* rev: CTCTTGGTGGCATACGAGAAAAT;

*FCGR2A* fw: GCTTCAACCATTGACAGTTTTGC;

*FCGR2A* rev: CCACGGGGGCTCAAGTTTC;

*GAPDH* fw: GAGTCAACGGATTTGGTCGT;

*GAPDH* rev: GACAAGCTTCCCGTTCTCAG;

*MMP2* fw: TACAGGATCATTGGCTACACACC;

*MMP2* rev: GGTCACATCGCTCCAGACT;

*MMP9* fw: AGACCTGGGCAGATTCCAAAC;

*MMP9* rev: CGGCAAGTCTTCCGAGTAGT;

*NOX4* fw: TGACGTTGCATGTTTCAGGAG;

*NOX4* rev: AGCTGGTTCGGTTAAGACTGAT;

*PAI1* fw: ACCGCAACGTGGTTTTCTCA;

*PAI1* rev: TTGAATCCCATAGCTGCTTGAAT.

The following mouse primers were used (Thermo Fisher Scientific, 5’→3’ orientation):

*Crp* fw: GTGCTGAAGTACGATTCATGGT;

*Crp* rev: CAATCCCCGTAGCAGACTCC;

*Gapdh* fw: AGGTCGGTGTGAACGGATTTG;

*Gapdh* rev: TGTAGACCATGTAGTTGAGGTCA.

### Protein isolation and Western blotting

HAoSMCs were lysed with ice-cold IP lysis buffer (Thermo Fisher Scientific) containing complete protease and phosphatase inhibitors cocktail (Thermo Fisher Scientific) [9,87]. Phosphorylation levels were determined 30 minutes and protein abundance 24 hours following treatments/silencing. Equal amounts of proteins were boiled in Roti-Load1 Buffer (Carl Roth) at 100ºC for 10 minutes, separated on SDS-polyacrylamide gels and transferred to PVDF membranes. The membranes were incubated with primary rabbit anti-RUNX2 (1:1000, #8486, Cell Signaling), rabbit anti-αSMA (1:1000, #19245, Cell Signaling), rabbit anti-phospho-p38 MAPK (Thr^180^/Tyr^182^) (1:1000, #9215, Cell Signaling), rabbit anti-p38 MAPK (1:1000, #9212, Cell Signaling), rabbit anti-Caspase 3 (1:1000, #9662, Cell Signaling), rabbit anti-CRP (1:1000, ab65842, Abcam), goat anti-FCGR2A (1:2000, AF1875, R&D Systems) or rabbit anti-GAPDH (diluted 1:5000, #2118, Cell Signaling) antibodies overnight at 4°C and then with secondary anti-rabbit HRP-conjugated antibody (diluted 1:1000, Cell Signaling) or anti-goat HRP-conjugated antibody (diluted 1:2000, Cell Signaling) for 1 hour at room temperature. The membranes were stripped in stripping buffer (Thermo Fisher Scientific) for 10 minutes at room temperature. Antibody binding was detected with ECL detection reagent (Thermo Fisher Scientific). Bands were quantiﬁed by using ImageJ software and the results are shown as the ratio of phosphorylated/ total protein/ GAPDH and of total protein/ GAPDH, normalized to the control group [9,88,91].

### Total antioxidant capacity

Total antioxidant capacity of HAoSMCs was measured in the cell lysate 24 hours after the treatment by using the colorimetric antioxidant assay kit (Cayman Chemical) [4,44,45]. Results are shown normalized to total protein concentration and to the control group.

### Calcification analysis

After 11 days of treatment, HAoSMCs were decalcified in 0.6 M HCl for 24 hours at 4°C and calcium content in the supernatant was determined by using QuantiChrom Calcium assay kit (BioAssay Systems). HAoSMCs were lysed with 0.1 M NaOH/ 0.1% SDS and protein concentration was measured by the Bradford assay (Bio-Rad Laboratories) and results are shown normalized to total protein concentration [4,11,70]. For Alizarin Red staining, HAoSMCs were fixed with 4% paraformaldehyde/PBS and stained with 2% Alizarin Red (pH 4.5). The calcified areas are shown as red staining [9,87].

### ALPL activity assay

ALPL activity in HAoSMCs was determined following 7 days of treatment by using the ALP colorimetric assay kit (Abcam) and the results are shown normalized to total protein concentration [69,70].

### Human samples

Human serum samples from patients with CKD from the CARVIDA subgroup of the GCKD study recruited in Erlangen, Germany [92] were used for the measurements. Serum calcification propensity was determined by a nephelometric method [83,87].

### Statistics

Data are shown as scatter dot plots and arithmetic means ± SEM and n indicates the number of independent experiments performed at different passages of the cells or the number of mice or human patients examined, respectively [4,70]. Normality was tested with Shapiro-Wilk test. Non-normal datasets were transformed (log, sqrt or reciprocal) prior to statistical testing to provide normality. Two groups were compared by unpaired two-tailed t-test. Statistical testing was performed by one-way Anova followed by Tukey-test (homoscedastic data) or Games-Howell test (heteroscedastic data). For correlation analysis, Spearman correlation test was performed. P<0.05 was considered statistically significant.

## Supplementary Material

Supplementary Figures
