# Supplementary material for: Impact of C-reactive protein on osteo-/chondrogenic transdifferentiation and calcification of vascular smooth muscle cells
Source: Aging (Albany NY). 2019 Aug 3;11(15):5445–62. doi: 10.18632/aging.102130 (PMC6710049; doi:10.18632/aging.102130)
Supplement: Supplementary Figures [file aging-11-102130-s001.pdf]

SUPPLEMENTARY FIGURES

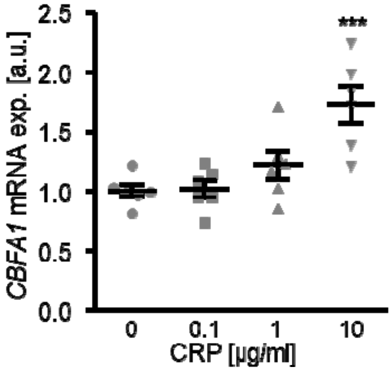

Supplementary Figure 1. CRP up-regulates *CBFA1* expression in HAoSMCs in a dose-dependent manner. Scatter dot plots and arithmetic means ± SEM (n=6; arbitrary units, a.u.) of *CBFA1* relative mRNA expression in HAoSMCs treated with the indicated concentrations of recombinant human CRP (0 - 10 µg/ml). ***(p<0.001) significant vs. control HAoSMCs.

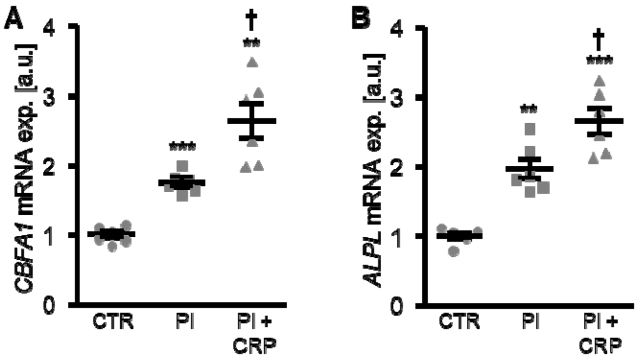

Supplementary Figure 2. CRP augments phosphate-induced osteo-/chondrogenic transdifferentiation of HAoSMCs. (**A, B**) Scatter dot plots and arithmetic means ± SEM (n=6; arbitrary units, a.u.) of *CBFA1* (**A**) and *ALPL* (**B**) relative mRNA expression in HAoSMCs treated for 24 hours with control (CTR) or β-glycerophosphate (Pi) prior additional treatment for 24 hours without and with 10 µg/ml recombinant human CRP. **(p<0.01), ***(p<0.001) significant vs. control HAoSMCs; †(p<0.05) significant vs. HAoSMCs treated with Pi alone.

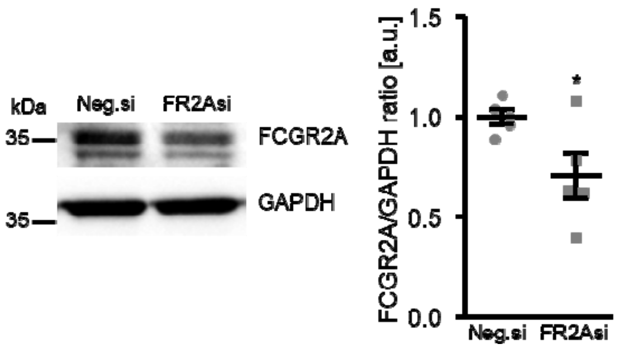

Supplementary Figure 3. Silencing efficiency of FCGR2A gene in HAoSMCs. Representative original Western blots and scatter dot plots and arithmetic means ± SEM (n=5; arbitrary units, a.u.) of normalized FCGR2A/GAPDH protein ratio in HAoSMCs silenced with negative control siRNA (Neg.si) or FCGR2A siRNA (FR2Asi). *(p<0.05) significant vs. Neg.si silenced HAoSMCs.

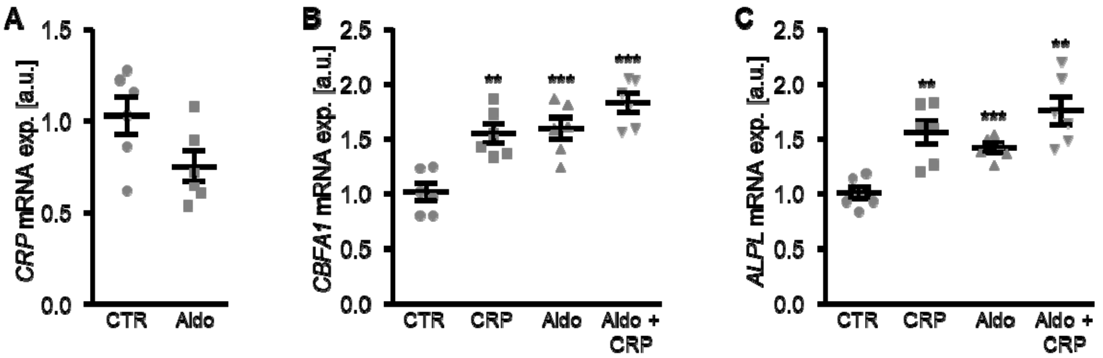

Supplementary Figure 4. *CRP* expression and CRP-induced osteo-/chondrogenic transdifferentiation of HAoSMCs are not significantly modified by aldosterone. (**A**) Scatter dot plots and arithmetic means ± SEM (n=6; arbitrary units, a.u.) of *CRP* relative mRNA expression in HAoSMCs treated with control (CTR) or 100 nM aldosterone (Aldo). (**B, C**) Scatter dot plots and arithmetic means ± SEM (n=6; a.u.) of *CBFA1* (**B**) and *ALPL* (**C**) relative mRNA expression in HAoSMCs treated with control (CTR) or 10 µg/ml recombinant human CRP without and with 100 nM aldosterone (Aldo). **(p<0.01), ***(p<0.001) significant vs. control HAoSMCs.
